# The potentials of probiotics on gluten hydrolysis; a review study 

**Published:** 2020

**Authors:** Najmeh Ramedani, Anousheh Sharifan, Fahimeh Sadat Gholam-Mostafaei, Mohammad Rostami-Nejad, Abbas Yadegar, Mohammad Javad Ehsani-Ardakani

**Affiliations:** 1 *Department of Food Science and Technology, Science and Research Branch, Islamic Azad University, Tehran, Iran *; 2 *Gastroenterology and Liver Diseases Research Center, Research Institute for Gastroenterology and Liver Diseases, Shahid Beheshti University of Medical Sciences, Tehran, Iran *; 3 *Foodborne and Waterborne Diseases Research Center, Research Institute for Gastroenterology and Liver Diseases, Shahid Beheshti University of Medical Sciences, Tehran, Iran*; 4 *Basic and Molecular Epidemiology of Gastrointestinal Disorders Research Center, Research Institute for Gastroenterology and Liver Diseases, Shahid Beheshti University of Medical Sciences, Tehran, Iran *

**Keywords:** Celiac disease, Gliadin, Gluten-free diet, Probiotics, Wheat

## Abstract

Celiac disease (CD) is an autoimmune disorder of the small intestinal mucosa in genetically susceptible subjects consuming gluten. Gluten in wheat, rye and barley is harmful for some individuals and leads to various symptoms. Research has shown that treatment with probiotics in CD patients could improve the symptoms by the gluten hydrolysis. For this purpose, different databases such as Medline, PubMed, Scopus, and Google Scholar were searched using the following keywords: Celiac disease, Wheat flour, Gluten, glutamine, Probiotic, Bifidobacterium, Lactobacillus, Enzymes, Wheat allergy, Immune system, T cells, HLA-DQ2, HLA-DQ8, Gluten-free diet, Proteolysis, α2-gliadin fragment, Gliadin, 33-mer peptide, and Zonulin. The search aimed to retrieve the articles published during 2000-2019. Today, a gluten-free diet (GFD) is the only celiac disease treatment. Biotechnological strategy based on probiotic treatment could degrade gluten. Research has shown that combination of the probiotic enzyme is more effective than single probiotic on gluten hydrolysis. The result of different studies showed that probiotic mixture has the capacity to hydrolyze a considerable concentration of the 33-mer of gliadin completely. The present study was aimed to investigate associations between the capacities of probiotics on gluten hydrolysis.

## Introduction

 Celiac disease (CD) is an intestinal malabsorption disorder, characterized by duodenal villous atrophy, caused by gluten proteins from wheat barley and rye, in people with genetic susceptibility (1,2). CD individuals may present gastrointestinal and non-gastrointestinal symptoms due to malabsorption (3,4). It is estimated that around 1% of the general population is affected by celiac disease that can affect both genders in all ages and races. Gluten-Free Diet (GFD) is the most effective treatment for these patients (5,6). Diet is the main factor in modulating the composition and function of gut microbiota, and intestinal microbiota plays a key role in determining a person's health status. In active CD patients, microbial compounds change and many studies have shown that probiotics are effective in repairing the composition of beneficial species (7). It is demonstrated that probiotics have gluten hydrolysis enzymes as an alternative or adjuvant treatment for relieving symptoms of CD and could be critical in the management of the disease (8,9). It seems that these enzymes can be used to digest and destroy gluten in patients with gluten sensitivity. The aim of this review article is to discuss characteristics of probiotics and the use of probiotics as a novel therapy for CD.

**Immunotoxic components of wheat**

Food allergy can be defined as an immune response for food (10). Wheat is the most common allergenic food and one of the eight most common food allergens (milk, eggs, fish, crustacean shellfish, tree nuts, peanuts, wheat, and soybean) (11). Wheat allergy is affected by immunologic responses to a range of proteins in wheat. These can be immunoglobulin E (IgE) mediated allergies, and non-IgE-mediated allergies, or a combination of both, as confirmed by other international guidelines of allergic reactions to wheat. Food allergens are usually proteins, but sometimes Heptane’s are known by allergen-specific immune cells and show specific immunologic responses (12). The main protein in wheat and some other cereals is called gluten. Gluten is obtained from cereals after the removal of the water-soluble components along with starch particles. It is the main insoluble protein from wheat, rye, and barley (Table 1) (9). 

**Table 1:** Protein present in cereals

Cereals	Protein
Wheat	Gliadin
Barley	Hordein
Rye	Secalin
Oat	Avenin
Maize	Zein
Rice	Glutelin

Proline and glutamine are the main gluten protein components, and enzymes in the intestine of healthy subjects can only partially digest gluten (13). In gluten-sensitive patients, the undigested protein fragments stimulate immunodominance with T-cells activation and proinflammatory interleukins. In CD patients, intestinal* immune response is triggered* by *gluten* protein and initiates the symptoms (14). One of the digestion resistant and immunodominant gluten peptides that are highly reactive to isolate celiac T-cells is α-gliadin 33-mer (15). Studies have shown that long-term use of gluten, at a rate of 10 to 50 mg per day, can damage the lining of the intestinal mucosa, by increasing the number of intrauterine lymphocytes (16,17). The U.S. Food and Drug Administration (FDA) announced that gluten free products containing no more than 20 ppm are safe for CD patients (18, 19). However, GFD is the only treatment for gluten-related disorders. Today, there are developing alternative therapies based on beneficial gut bacteria (probiotics, such as *Bifidobacterium* and *Lactobacillus*)(20). Wheat gluten in food could be* remove*d or reduced by biotechnological strategies with probiotics that hydrolyze immunogenic gluten component.

**The role of probiotics in gluten hydrolysis**

Recent advances in the treatment of CD provides new and promising strategies. Therefore, other treatments have been introduced, like genetically modified gluten and gliadin, tissue transglutaminase inhibitors, zonulin inhibitors and lately probiotics (21). The FAO/WHO reports that probiotics are living microorganisms in foods or supplements that are administered in adequate amounts to improve the host’ health (21,22). Nowadays, numerous probiotics have been advised as adjuvant therapy for controlling the CD (23). A novel treatment for CD is microbial proteases which are efficient in detoxifying gluten. Clinical research and further therapeutic interventions showed that the probiotic mixture was more effective than a single strain for CD (8,24). The relative abundance of beneficial microbes by probiotics is one of the best options for CD therapy (Table 2). The most important probiotics used in foods and supplements are *Bifidobacteria* and *Lactobacilli* strains. *Bifidobacterium lactis *can stimulate specific antigen-specific cytotoxic T lymphocytes, natural lethal cells and macrophages (9). Probiotics can be used to destroy the epitopes before reaching the intestinal mucosa, repairing epithelial healing, or directly targeting pathological immune responses (22,25). Dipeptidyl peptidase is often used for gluten hydrolyzation. Probiotics secrete the enzyme exo-peptidase to break the end of a peptide. A report showed an association between lower intestinal dipeptidyl peptidase activity and mucosal damage in celiac patients and other malabsorption syndromes (26). One way to reduce the risk of gluten contamination of gluten-free products is to ferment the dough with lactic acid bacteria (27). 

**Table 2:** Mechanism of probiotics in celiac disease

Mechanism	Possible probiotics
Enzymatic gluten degradation or pre-ingestion fermentation	VSL#3 long-lasting fermentation by Lactobacilli and fungal proteases
Maintenance of barrier of gastrointestinal tract	Bifidobacterium and Lactobacilli play a fundamental role

**Figure 1:**
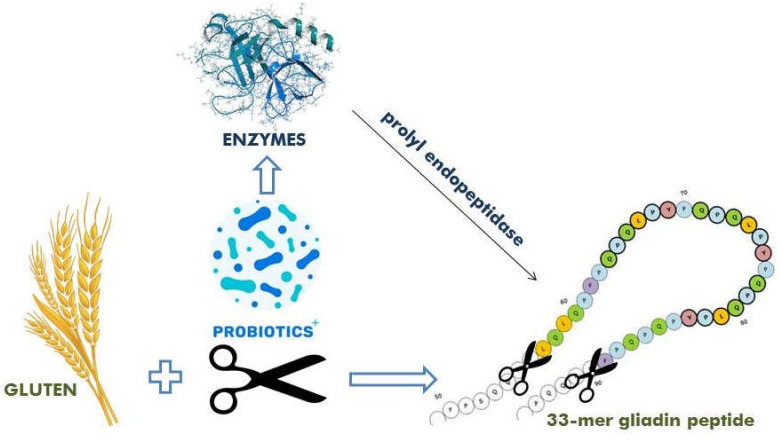
Hydrolysis of 33-mer peptide with exposure to probiotics and their enzymes such as prolyl-endopeptidase

A combination of probiotics with specific properties appears to be effective in gliadin hydrolysis than single strains (24).

Probiotics can impress the CD by three mechanisms. The first is to digest gluten proteins into small unsafe polypeptides, removing or reducing the CD pathogenesis, hence stopping immune reactions. The second is to protect the intestinal barrier by barring the availability of immunogenic polypeptides to lamina propria. The third and the most interesting is the critical role of probiotics in the intestinal microbiota hemostasis and the regulation of the innate and adaptive immune system (20). The possible mechanism for using enzyme therapy for celiac patients is that degraded gluten proteins in the small intestine contain gliadin peptides, which are rich in proline, glutamine, and 33 mer, which can be broken down by enzymes in the gastrointestinal tract. Therefore, this can be considered as a substitute to GFD (15). Endopeptidase enzyme in probiotic could separate and hydrolyze the internal bonds of gliadin. This enzyme can digest gluten peptides into small and safe molecules, which are well tolerated in celiac patients (28). 33-mer gliadin peptide from α2 gliadin is the main immunogenic component for CD and other gluten-related diseases (15). The α-gliadin 33-mer is a digestible gluten-resistant peptide, and a structural change of 33 mer could be the first unknown cause of the disease (Fig. 1). The enzyme activity of probiotics can break down peptide bonds including proline.

**Previous research about the use of probiotics for gliadin hydrolysis**

In a recent randomized controlled trial in subjects with non-celiac gluten sensitivity, 37 adult patients were enrolled in the study, 12 were treated with enzyme combination and 25 with placebo. The enzyme mixture was consumed 3 times a day for a month. This study showed a statistically significant decrease in the level of IgA anti-gliadin antibodies and inflammation compared to placebo group. Only one CD patient was positive for gluten IgG after enzymatic treatment (29). Recently, food technicians have used a gluten hydrolysis strategy with a residual concentration of less than 10 ppm, which is a diet with lactobacilli and fungal proteases (29,30). In another study, the effect of bacteria on the destruction of gliadin-induced cells by probiotic bacteria (fermented and *Bifidobacterium lactis*) in intestinal epithelial cell culture was determined. They suggested that *Bifidobacterium lactis* was able to protect the epithelial cell permeability caused by gliadin. Furthermore, both probiotics were able to protect against cell ruffling and alterations in tight junctions. Also, probiotics without gliadin as a control did not show significant changes to the intestinal epithelial cells. Probiotics can improve the damage of intestinal epithelial cells caused by gluten contaminated foods and may even accelerate mucosal healing after the initiation of a GFD (31). Studies conducted in 2015 showed that the gliadin peptides may reduce by the activity of proteinase and peptidase in the gut microbiota, which in turn affect their toxicity. (26,32). Several studies demonstrated that 144 strains of 35 bacterial species can hydrolyze gluten. Most of these strains were from *Firmicutes* and *Actinobacteria *phyla that can improve CD symptoms. A total of thirty-one strains of gluten-degrading bacteria were isolated from human small intestine, 27 of which showed peptidolytic activity compared to 33 mer peptides (28,33). Researchers have developed a new method for reducing gluten allergy by making sourdough with probiotics such as *Lactobacillus* to cleave proline and gliadin peptides, including the 33-mr peptides (15, 33). Most of these cases are *lactobacilli* which can reduce the immunogenicity of the 33-mer peptide and provide a protective effect for *lactobacillus* in gluten hydrolysis (20). *Lactobacilli* need large amounts of amino acids nitrogen for suppling metabolic energy and growth. The mixtures of *lactobacilli* and *Bifidobacteria* have a complex proteolytic and lipolysis effect that can be involved in the breakdown of gluten (gliadin) and its peptides. These are used as a probiotic supplement for the treatment of celiac disease (20,34). A few other probiotic compounds are used to treat CD, including Florisia (*Lactobacillus brevis, Lactobacillus plantarum, Lactobacillus salivarius, and subsp. Salicinius*), Oxadrop (*Lactobacillus acidophilus, B. infantis, L. brevis, and S. thermophilus*), and Yovis (*B. Infantis, B. breve, L. acidophilus, B. longum, L. plantarum, L. casei, L. delbrueckii* *subsp*. *Bulgaricus, Thermophilus, Streptococcus salivarius subsp* , *Enterococcus faecium* and the most beneficial probiotic is the mixture of eight strains (VSL#3), *Bifidobacterium breve, Bifidobacterium infantis, acidophilus, Lactobacillus plantarum, Lactobacillus casei, Lactobacillus delbrueckii sub sp. bulgaricus, Streptococcus thermophilus and Bifidobacterium longum*, which decreased wheat sensitivity. The results showed that VSL#3 c is more effective on gliadin degradation than a single strain alone, and had a beneficial effect on the treatment of CD (24). A research by Shan et al. showed that unique 33 amino acid peptides out of 266 amino acid of α2 gliadin are resistant to hydrolyze in the gastrointestinal tract. It showed that an oral bacterial peptidase could be used to detoxify the predominantly immunosuppressive gliadin epitopes (35). A study in 2008 used the mixture of bacteria and barley-derived proteases-PEP and endoprotease B-isoform, respectively and showed that oral consumption of this mixture by celiac patients can change the gluten epitopes to non-toxic parts; therefore, they proposed that these protease enzymes may be a useful treatment and might allow CD patients to take in modest amounts of gluten in their diets (36). An earlier study showed that epithelium with exposure to probiotics in gastrointestinal tract can hydrolyze gluten, which is the one way to maintain the health of CD patients. The *lactobacilli strain hydrolyzed gluten* under the *simulated gastrointestinal* condition and the gluten content was lower than 10 ppm after six hours (8). Previous studies have shown significant alteration of intestinal permeability in Caco-2 cells when the *Lactobacillus rhamnosus* was added to gliadin peptides, all demonstrating the inhibitory effect on pathogenic bacteria through interaction with lymphatic tissue and villi (37). 

Bifidobacterium strains such as *Bifidobacterium longum and Bifidobacterium bifidum* reduce the toxicity and inflammatory factors of gluten peptides. Also, they have potentials to improve CD symptoms (24,38). Various studies have shown that the intervention of probiotics may offer new treatment including (24), gluten vaccination (39), gluten tolerance and immunomodulation (40), tissue transglutaminase inhibitors (41,42), HLA-DQ2 or HLA-DQ8 blockers (43), genetically modified gluten (44,45), and glutenase supplement diet (16). These possible treatments are available for CD management but require further research (9). A new and safe treatment of celiac disease is cleaving the gliadin into small nontoxic peptides before reaching the intestinal mucosa, such as the use of oral supplements with prolyl oligopeptidase (46). A study evaluated the effect of aspergillopepsin from *Aspergillus niger* and dipeptidyl peptidase from *Aspergillus oryzae* showed that gluten hydrolyzing simulated intestinal conditions. The *experiments demonstrated that* the use of single peptidase alone (neither aspergillopepsin nor dipeptidyl peptidase) cannot eliminate gluten peptides and the use of enzyme combinatin can significantly reduce gluten levels (47). In a study conducted in 2015, *Aspergillus niger* prolylendoprotease enzyme enhanced gluten degradation in the stomach of healthy subjects (48). Many bacterial and fungal enzyme supplements can break down gluten and prolamins with their endopeptidases or proteinases (49). Nowadays, the effective treatment available for CD individuals is a forceful lifelong GFD (45). Although GFD increases the absorption of vitamins and nutrients, a gluten-free diet can prevent severe autoimmune diseases caused by celiac disease. Unfortunately, even in strict diet plans, there may be a small amount of gluten in the GFD and many people inadvertently eat gluten-containing foods (36). Also, CD patients have issues regarding availability, quality and variety of gluten-free products’ where these products are generally more expensive than their counterparts (50). Deep understanding of biochemical and molecular mechanism through probiotics that affect CD will help formulate probiotics and their optimum operational conditions such as time, temperature and concentration which enhance gluten hydrolysis create a novel therapeutic model to change the course of CD (20). 

## Conclusion

 Celiac disease is a well-known autoimmune disorder of the last decades. Nowadays, a lifelong strict gluten-free diet (GF) is an effective treatment for CD and scientists are looking for beneficial treatments to improve the quality of life in these patients. Furthermore, in the last two decades, probiotics have been increasingly consumed for their beneficial effects. It is proposed that a combination of probiotics can completely hydrolyze the toxic and allergenic components of gluten by their enzymes, including 33 mer degradation of gliadin, and the enzymes can revolutionize the gluten-free manufacturing industry. In the future, GFD alternatives, which are the only effective therapies available, can be used as enzyme supplements as a new treatment strategy for CD instead of using a gluten-free diet. 

## Conflict of interests

The authors declare that they have no conflict of interest.
